# Diffuse idiopathic skeletal hyperostosis as a cause of progressive dysphagia: a case report

**DOI:** 10.1186/1757-1626-1-416

**Published:** 2008-12-23

**Authors:** Constantine Constantoyannis, Theodore Papadas, Demetrios Konstantinou

**Affiliations:** 1 Department of Neurosurgery, University Hospital of Patras, Patras, Greece; 2 Department of Head and Neck Surgery, University Hospital of Patras, Patras, Greece

## Abstract

**Background:**

Forestier's disease, also known as diffuse idiopathic skeletal hyperostosis (DISH), is an idiopathic rheumatological abnormality in which exuberant ossification occurs along throughout the body, but most notably the anterior longitudinal ligament of the spine.

**Case presentation:**

We report on a 75-year-old white patient with progressive difficulty in swallowing and dysphagia, resulting in weight loss over the last two years. Radiological evaluation, (x-rays and Magnetic resonance imaging), confirmed the diagnosis of DISH, and revealed marked compression of the esophagus at the C5-6 level, due to excessive ossification of the anterior longitudinal ligament of the cervical spine.

The patient was treated with anterior cervical approach for removal of the hyperostosis without fusion. He had marked improvement in swallowing function and was able to resume a normal diet after one month.

**Conclusion:**

Diffuse idiopathic skeletal hyperostosis or Forestier's disease is an uncommon etiology of difficulty in swallowing and progressive dysphagia. Surgical excision of the cervical osteophytes typically leads to excellent symptomatic results.

## Background

Mosher in 1926 was the first who reported dysphagia due to a cervical spine osteophyte [1]. Iglauer reported the first surgical excision of a cervical spine osteophyte producing dysphagia in 1938 [2]. In 1950 Forestier and Rotes-Querol described an ankylosing disease of the spine developing in elderly people [3]. This entity was known as Forestier's disease. In the 1970s, Resnick termed this condition Diffuse Idiopathic Skeletal Hyperostosis (DISH) [4,5]. He insisted upon three strict radiographic features of the spine as a prerequisite for diagnosis: (*a*) flowing calcification and ossification within the anterior longitudinal ligament involving at least four contiguous vertebral bodies, most commonly those of the thoracic spine; (*b*) a minimal degree of degenerative disc disease; and (*c*) absence of apophyseal joint ankylosis and sacroiliac erosions, sclerosis, or intraarticular osseous fusion [5]. Predominant among the pathological entities that can be confused with DISH are osteophytes accompanying degenerative disease of the cervical spine, and ankylosing spondylitis.

DISH is not uncommon disorder among rheumatological patients and has been reported in 12% of random autopsy series in a Veterans Administration hospital population [4,5]. However, due to the bone projection away from the spinal cord, it is rare for a patient to have a symptomatology that would elicit evaluation by a neurosurgeon. Although these patients are typically asymptomatic, there is documentation of DISH patients presenting with spinal instability, upper gastrointestinal, respiratory, and neurological problems [2,6-8]. Resnick et al. also found a 17 to 28% incidence of dysphagia secondary to cervical hyperostosis in patients with DISH, and surgical intervention via an anterior cervical approach was required in 8% who failed to respond to conservative treatment [5].

## Case presentation

**Figure 1:**
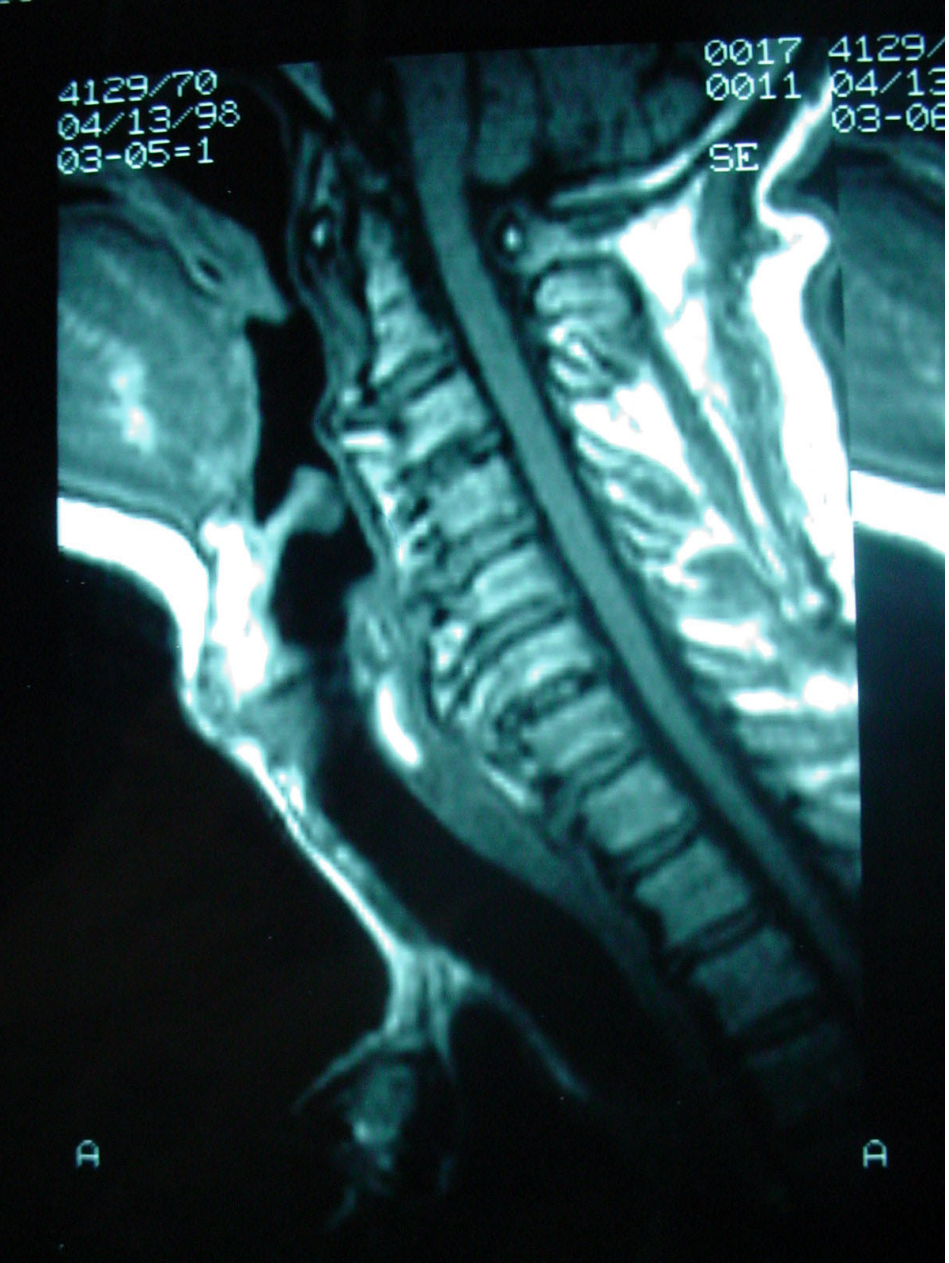
**Axial MRI of the cervical spine revealed an elongated ossification of the frontal planes of the vertebral bodies**.

**Figure 2:**
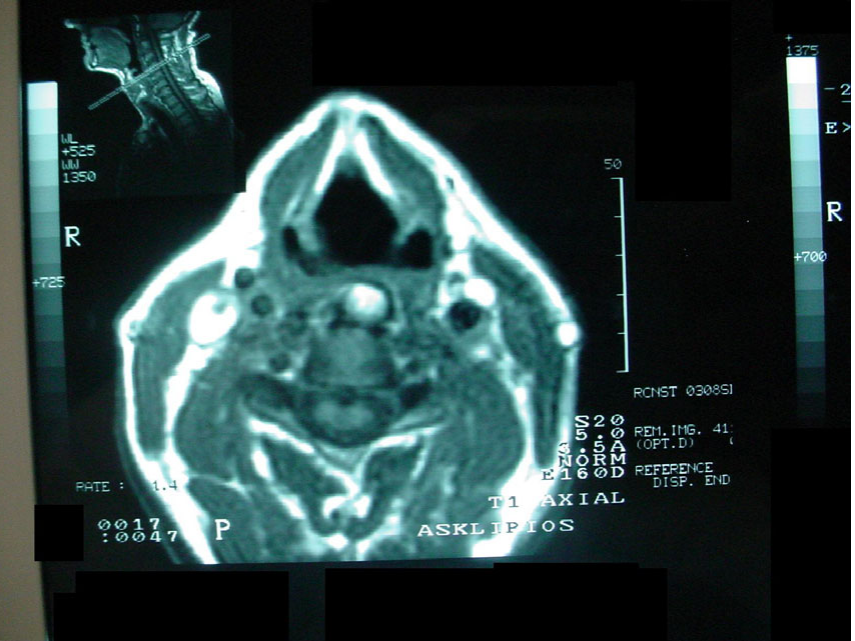
**Sagittal MRI of the cervical spine showing the abnormal prevertebral tissue formatted by the osteophytes**.

**Figure 3:**
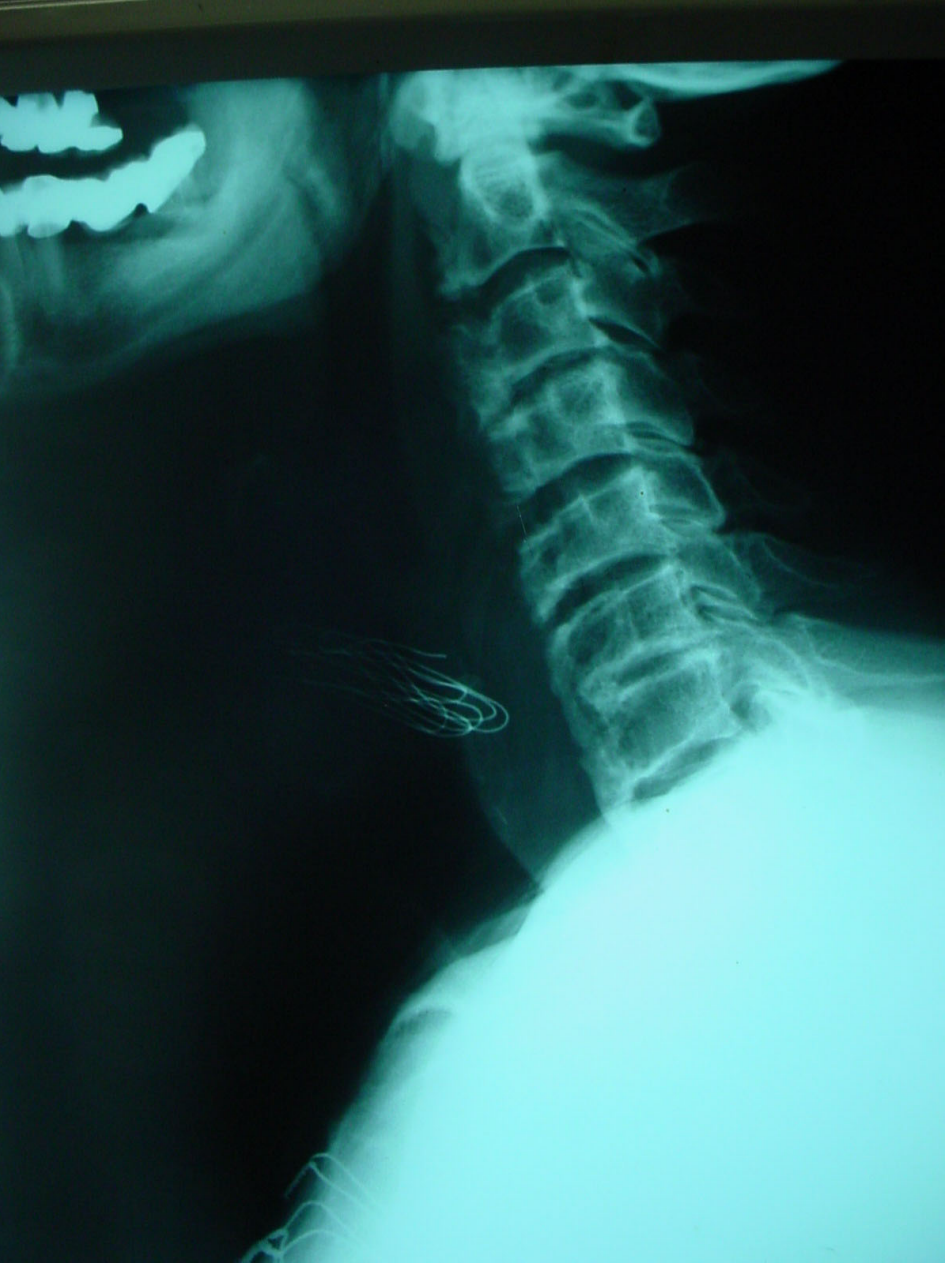
**Postoperative lateral cervical spine x-rays showed absence of the osteophytes and normalization of the normal structure**.

A 75-year-old white man was seen at University Hospital of Patras with the main complain of increased difficulty in swallowing solid food over the past two years. He denies any symptoms suggestive of cervical radiculopathy or myelopathy. He also denies symptoms of neck pain or discomfort. Over the past six months he had altered his diet to include only soft foods and liquids and he had an approximately 10 kgr weight loss over that period of time. His medical problem included hypertension, adult-onset diabetes mellitus, coronary artery disease, and hyperlipidemia. On physical examination, the patient was alert, afebrile, and well oriented, with stable vital signs. The neck had significantly decreased mobility, but no apparent mass was present. Routine cervical spine films revealed prodigious osteophytes of the cervical spine involving the bodies of C-2, C-3, C-4, C-5, C-6, C-7, and T-1 consistent with DISH. A magnetic resonance imaging (MRI) scan (Fig. 1) and a computerized tomography (CT) (Fig. 2), of the cervical spine revealed an elongated ossification of the frontal planes of the vertebral bodies, especially at the height of the C 5–6 levels, with elongated and spiky spurs projecting into the soft tissues of the neck. The patient underwent uneventful operative excision of the anterior cervical osteophytes through a routine anterior-lateral approach. He had marked improvement in swallowing function and was able to resume a normal diet after one month. Postoperative cervical spine MRI and x-rays showed absence of the osteophytes and normalization of the normal anatomical structure of the pre-vertebral tissues (Fig. 3). There was no evidence of cervical instability, either clinically or on routine x-rays.

## Discussion

The etiology of DISH is unknown. Some data suggest that ossification of ligaments is an attempt to establish rigidity and limit motion [9]. Radiographic series demonstrate that calcification of the anterior longitudinal ligament in the cervical spine continues until movement is eliminated across adjacent motion segments [10]. In addition, recurrent ossification accompanied by dysphagia has been reported following resection of osteophytes along the cervical spine [11]. However the thoracic region is the most immobile segment of the spine and the most common site for ossification of the anterior longitudinal ligament in DISH [5]. This argues against mobility being the only catalyst for ossification. The most frequent level of involvement related to dysphagia is C 5–6 followed by C 4–5, C 2–3 being the least common level affected [12]. This condition occurs more frequently in men than women, typically in their 60's [3]. Once the anterior osteophyte is large enough to compress or displace the esophagus and/or the trachea, the patient may complain of dysphagia, odynophagia, dysphonia, a sensation of a foreign body in the throat, or a constant urge to clear the throat. Aspiration of liquids or solids, airway obstruction, stridor, and obstructive sleep apnea has been also reported as presenting symptoms of cervical osteophytosis [13]. Dysphagia is characteristically greater for solids than for liquids. Aspiration may be greater for liquids than for solids. Foreign body sensation may be by mucosal abrasion as food passes over a protruding osteophyte. Odynophagia may result from hypo pharyngeal ulceration at a point of pressure between the posterior cricoid cartilage and a protruding osteophyte. Dysphonia or airway obstruction may result from laryngeal edema, arytenoids ankylosis, or vocal cord paralysis caused by an osteophyte at the cricoid level. Obstructive sleep apnea and stridor may result of impingement of the osteophyte on the laryngeal vestibule [4,5,13]. It is unclear how cervical osteophytes cause dysphagia; however, several possible explanations have been advanced: (a) a osteophyte may cause dysphagia by simple mechanical obstruction; (b) osteophytes may also cause dysphagia if they are located opposite a fixed point of the esophagus such as the cricoid cartilage (C 6 level); (c) inflammation in the immediate vicinity of the osteophyte; (d) pain and spasm. Most probably a combination of some or all of these circumstances is responsible for dysphagia in many cases [14].

Evaluation of patients with progressive dysphagia should include routine otolaryngologic examination, cervical spine films, cervical MRI and video fluoroscopic swallowing study. On examination, the trachea may be displaced from the midline with a hard palpable mass between the soft tissue of the sternocleidomastoid laterally, and the trachea and esophagus medially [14]. Although some clinicians have recommended endoscopy, this may in fact prove dangerous and has been a common cause of esophageal perforation [15]. Differential diagnosis includes esophageal tumors, esophageal stricture, Zenker's diverticulum, motility disorders, Plummer-Vincent's syndrome and other mediastinal mass lesions [12]. Controversy appears in the literature over the appropriate treatment of dysphagia due to Forestier's disease. Several authors recommend only observation or diet modification and a regimen of anti-inflammatory medications [13]. However, for progressive dysphagia, surgical excision of the anterior cervical osteophytes is recommended. Concomitant cervical fusion or discectomy are not felt to be necessary. Large osteophytes also present a risk of esophageal injury during the operative exposure. The esophagus may be difficult to mobilize and somewhat adherent to other cervical fascia due to local inflammatory reaction. Re-ossification with new osteophyte formation may rarely occur and repeat operation may be indicated if dysphagia symptoms return [10].

## Conclusion

Diffuse idiopathic skeletal hyperostosis or Forestier's disease is an uncommon etiology of progressive dysphagia. Anterior cervical excision of the osteophytes typically leads to excellent symptomatic results.

## Consent

Written informed consent was obtained from the patient for publication of this case report and accompanying images. A copy of the written consent is available for review by the Editor-in-Chief of this journal.

## Competing interests

The authors declare that they have no competing interests.

## Authors' contributions

All authors' contributed the same.
